# Colony size predicts division of labour in attine ants

**DOI:** 10.1098/rspb.2014.1411

**Published:** 2014-10-22

**Authors:** Henry Ferguson-Gow, Seirian Sumner, Andrew F. G. Bourke, Kate E. Jones

**Affiliations:** 1 School of Biological Sciences, University of East Anglia, Norwich Research Park, Norwich, Norfolk NR4 7TJ, UK; 2 Institute of Zoology, Zoological Society of London, Regent's Park, London NW1 4RY, UK; 3 Centre for Biodiversity and Environment Research, Department of Genetics, Evolution and Environment, University College London, Gower St., London WC1E 6BT, UK

**Keywords:** Formicidae, queen–worker dimorphism, worker size polymorphism, social evolution, caste evolution

## Abstract

Division of labour is central to the ecological success of eusocial insects, yet the evolutionary factors driving increases in complexity in division of labour are little known. The size–complexity hypothesis proposes that, as larger colonies evolve, both non-reproductive and reproductive division of labour become more complex as workers and queens act to maximize inclusive fitness. Using a statistically robust phylogenetic comparative analysis of social and environmental traits of species within the ant tribe Attini, we show that colony size is positively related to both non-reproductive (worker size variation) and reproductive (queen–worker dimorphism) division of labour. The results also suggested that colony size acts on non-reproductive and reproductive division of labour in different ways. Environmental factors, including measures of variation in temperature and precipitation, had no significant effects on any division of labour measure or colony size. Overall, these results support the size–complexity hypothesis for the evolution of social complexity and division of labour in eusocial insects. Determining the evolutionary drivers of colony size may help contribute to our understanding of the evolution of social complexity.

## 1. Introduction

Insect eusociality represents one of the major transitions in evolution [1–3]. In these events, groups of formerly free-living individuals become sufficiently integrated to be considered individuals in their own right. A key component of this process is the evolution of division of labour [1,4,5]. In eusocial societies, the presence of a sterile caste (workers) and a dedicated reproductive caste (queens) creates a reproductive division of labour, while behavioural or morphological specialization within the worker caste on tasks such as brood care, nest maintenance, foraging and defence creates a non-reproductive division of labour. In ‘simple’ eusocial societies, queens are morphologically similar to workers, and workers are monomorphic. In ‘complex’ eusocial societies, queen–worker dimorphism is extreme and there is wide variation in worker size, often accompanied by discrete physical worker castes [4,6,7]. Previous studies have suggested positive effects of division of labour on the foraging efficiency and colony productivity of social insects, and hence on their ecological success [8–11]. However, the evolutionary determinants of division of labour have been less well researched.

The ‘size–complexity hypothesis’ proposes that, as colony size increases, workers and queens maximize their inclusive fitness by specializing in non-reproductive and reproductive roles, respectively [4,6,12,13]. As such specialization permits further increases in colony size, the degree of non-reproductive and reproductive division of labour both increase via positive feedback between social complexity and colony size. The hypothesis therefore leads to the prediction that colony size is positively associated with two key aspects of social complexity—non-reproductive and reproductive division of labour.

Although theoretical models [14,15] and single taxon experimental studies [16–18] offer some support for the size–complexity hypothesis, whether the predicted across-species relationships occur remains unclear, as early comparative studies [6,19] were informal and lacked an explicit evolutionary framework [20]. More recent phylogenetic comparative studies across formicoid ant species [21] and corbiculate bees [22] found positive correlations between colony size and measures of social complexity. While informative, these studies either omitted species with very large colony sizes (10^6^ workers or more) [21], potentially missing the predicted relationships [4], or measured social complexity as a single variable [22], potentially missing the independent effects of colony size on individual components of social complexity, namely the extent of reproductive and non-reproductive division of labour [21].

Moreover, no previous study has considered possible effects of environmental factors, yet these also potentially influence the relationship between colony size and division of labour. For example, in ants, a nonlinear relationship exists between colony size and primary productivity such that higher primary productivities are associated with decreasing colony size [23]. This suggests that it is important to control for environmental factors when analysing correlates of colony size across species. Environmental factors may also influence division of labour directly. Experiments show that in the desert ant *Cataglyphis velox*, smaller workers forage at lower temperatures than larger ones, suggesting that worker size variation has evolved as a mechanism for colonies to cope with diurnal fluctuations in temperature [24]. Overall, therefore, the potential role of environmental factors needs to be considered to gain a full understanding of the evolution of division of labour.

To test for evolutionary relationships between division of labour, colony size and environmental factors, we gathered species-specific data on social traits and evolutionary relationships and conducted a phylogenetically controlled comparative analysis within the neotropical ant tribe Attini. We used worker size variation and queen–worker dimorphism as measures of non-reproductive and reproductive division of labour, respectively. We selected ants as the focal taxon because ants are the most socially diverse and ecologically successful social insect group [7,25]. We focused on the tribe Attini because this taxon (252 species) exhibits wide variation in worker size, queen–worker dimorphism and colony size, and occurs in a relatively broad range of habitats and latitudes [26–32]. In addition, the Attini are predominantly monogynous [26], i.e. having a single queen heading a colony, such that the size–complexity hypothesis can be tested in the absence of confounding effects of variation in colony genetic and social structure brought about by polygyny (multiple queens heading colonies) [4,33]. Controlling for environmental variation, we show that evolutionary increases in colony size across the Attini are associated with increases in both worker size variation and queen–worker dimorphism.

## 2. Material and methods

### (a) Data collection

We used all Attini genus names, including synonyms, as search terms in Web of Knowledge, Scopus and Google Scholar literature databases up to 2013. Literature sources resulting from this search were scanned manually and relevant data were extracted. Data from secondary sources were excluded. Additional data were collected from AntWeb (http://www.antweb.org). Data from 58 sources covering 632 observations of populations for 57 out of a total of 252 species in the Attini were collected (see the electronic supplementary material, table S1). These data represented all attine genera (except for the socially parasitic *Pseudoatta,* a derived form of *Acromyrmex* [34], and the recently erected genus *Paramycetophylax*). Taxonomic names followed the Bolton World Catalogue (http://www.antweb.org).

Data were collected and stored in a database following recommendations in Kattge *et al*. [35]. The following traits were recorded: worker and queen size measured as head width in millimetres (92 observations of populations for 36 and 39 species for worker and queen head widths, respectively), colony size (number of workers at colony maturity) (178 observations, 43 species) and geographical location (362 observations, 48 species). Where specific coordinates were not supplied in the source reference, they were inferred from the description of the locality except where the specified area exceeded 20 km^2^. In these cases, the locality was deemed to be uninformative and excluded from analysis. Head-width measurements taken from AntWeb (http://www.antweb.org) (17 and 13 species for worker and queen head widths, respectively) were measured using the image analysis software ImageJ [36]. To ensure the measurements obtained from the specimens on AntWeb were representative, we compared the measurements obtained from images of seven species well represented both in the literature and on AntWeb. In all cases, the AntWeb measurements were not significantly different from those obtained from the literature (paired *t*-test, *t* = 1.044, *p* = 0.34, *n* = 7).

We calculated per-species means for colony size and worker and queen head width (see the electronic supplementary material, table S1) by averaging the mean value from each observation weighted by its sample size as
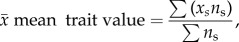
where *x_s_* is the mean of the observation, *n*_s_ is the observation sample size and ∑*n*_s_ is the sum of all sample sizes of the observations contributing to the per-species mean for each trait. Observation sample sizes ranged from 1 to 1016; however, in many cases, observation sample sizes were not given in the original source and here we assumed it to equal 1. We report ∑*n*_s_ as the sample size for each per-species mean trait value as this is more appropriate to the nature of our data than the number of sources.

To measure non-reproductive division of labour for each species, we quantified worker size variation using the coefficient of variation in worker head width (36 species) following previous authors [21]. We selected the coefficient of variation as it was an objective measure of trait variation that avoided subjective assessment of the number of discrete worker castes. In addition, using number of worker castes to measure worker size variation would not quantify non-reproductive division of labour correctly in species with size-based polyethism and a continuous distribution of worker sizes [8,9]. Worker size variation was calculated as
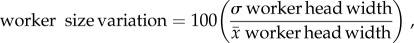
where 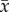 = mean and *σ* = standard deviation. Standard deviation of worker head width was calculated as the standard deviation of all mean worker head width observations contributing to each per-species value, and 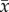 worker head width was calculated by averaging the mean value from each observation weighted by its sample size. Our measure of worker size variation was not influenced by sample sizes: a linear regression model (for data where observation sample sizes were known, controlling for study effort) of square-root worker size variation and log ∑*n*_s_ was not significant (log ∑*n*_s_, *β* = 0.002, d.f. = 2, 30, *p* = 0.857).

To measure reproductive division of labour for each species, we quantified queen–worker dimorphism as the percentage difference between mean queen head width and mean worker head width (30 species), that is, as
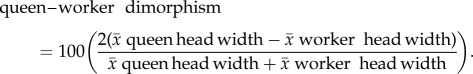


For both measures, we selected head width as a measure of body size because it is the most commonly reported measure of queen and worker size in the literature and, although showing allometric variation in some cases (e.g. *Atta* [37]), it correlates well with body size [7,38–40].

To quantify environmental variation, we downloaded the following data layers from the online database BioClim (http://www.worldclim.org/bioclim): diurnal temperature range, isothermality, temperature seasonality and precipitation seasonality. We resampled BioClim data from its original resolution into a grid size of 10 arcmin per pixel (approx. 20 km^2^ at the equator) to reflect the threshold at which we discarded locality information. The R package ‘raster’ [41] was used to extract these environmental values for sets of coordinates derived from the source references for each ant species, and mean values for each species were calculated for use in subsequent analyses (48 species). Species locations ranged from latitudes between 41.0° (DEC) and −29.7° (DEC), showing a broad range of environmental variation (see the electronic supplementary material, table S1 and figure S1).

### (b) Phylogenetic reconstruction

Analyses of traits across species are often confounded by non-independence because closely related taxa have similar traits due to shared evolutionary history [42]. This non-independence can be statistically controlled for in analyses by incorporating an estimate of evolutionary relatedness. However, constructing rigorous and unbiased estimates of evolutionary relationships for all the taxa of interest is challenging when existing phylogenetic studies are incomplete and conflicting and use non-overlapping datasets [43]. Previous phylogenetic analyses of social traits in ants have not used formal methods to link separate phylogenies or cover missing taxa [21,44,45], resulting in phylogenetic estimates that may be biased and that contain no estimates of uncertainty.

Here, we go beyond previous studies and construct an Attini consensus phylogeny that analyses the available phylogenetic hypotheses to generate a new, unbiased estimate, accompanied by calculations of uncertainty. We constructed a phylogeny using supertree protocols [43,46,47], because these methods allowed us to produce a tree that maximized the number of species in the resulting phylogeny and therefore the phylogenetic overlap with the species in our trait dataset. Available phylogenetic information for Attini is mainly based on morphological characters and is not well represented by genetic sequences in GenBank. As other consensus phylogenetic methods rely on constructing an estimate from genetic sequences (e.g. the supermatrix approach [48]), we chose the supertree method as the most appropriate for these data as it can combine both morphological and genetic evidence. We used matrix representation with parsimony (MRP) [43,46]. This method involves coding the topologies of published phylogenies into a weighted character matrix that is analysed using maximum parsimony to produce a composite tree [49]. MRP was selected for consistency with previous studies employing supertree methods [46,50] and has been shown to return trees as well supported as those derived using other methods [51–53]. Prior to analysis, we implemented safe taxonomic reduction [54] to remove species that had little or no phylogenetic signal, which if retained would reduce the resolution of the final tree. The final matrix had 71 out of 252 species drawn from 12 source phylogenies (see the electronic supplementary material, table S2), representing all genera of Attini (except for *Paramycetophylax*). We implemented a parsimony ratchet [55] in PAUP* v. 4.0b10 [56] to analyse the matrix and took the resulting consensus. Support values for each node of the tree were generated using rQS [57], which prunes the supertree and each source tree to confer identical taxon sets on them and then compares the topologies, assigning each node a score between +1 (full support) and −1 (total conflict). Positive rQS values indicate support for a node. We obtained, aligned and concatenated 4321 bp of sequence data for five genes (*wingless*, *long-wavelength Rhodposin*, *elongation factor 1 alpha 1*, *elongation factor 1 alpha 2* and *cytochrome oxidase subunit 1*) from species of the Attini represented in GenBank [58]. We used the software packages BEAST [59] in conjunction with the alignment to calculate relative branching time estimates for the species shared between the alignment and the supertree following previous studies [46] under a strict molecular clock [60]. Three Attini fossils were used as calibration points at nodes 11 [61], 50 [62] and 54 [63] and a non-Attini fossil (*Pheidole*) [64] was used to date node 1 (see the electronic supplementary material, figure S2), allowing dates to be calculated from relative branch lengths. The perl script chronographer.pl [65] was used to infer missing node ages based on a pure-birth model resulting in a supertree topology with branching time estimates following [46]. The final supertree was deposited in TreeBASE (http://purl.org/phylo/treebase/phylows/study/TB2:S14540).

### (c) Data analysis

We tested all social traits for phylogenetic signal using the phylogenetic generalized least-squares (PGLS) function of the R package ‘caper’ [66]. All traits contained phylogenetic signal (worker size variation *λ* = 0.97, queen–worker dimorphism *λ* = 0.94 and colony size *λ* = 0.91), and so we used phylogenetically controlled regression models in subsequent analyses.

Data were checked for normality and outliers. We used a square-root transformation for worker size variation and a natural log transformation for queen–worker dimorphism and colony size to normalize the data. We checked for colinearity in all models separately by calculating variance inflation factors (VIF) for each covariate. Covariates were sequentially eliminated starting with the largest VIF until all VIFs were less than three [67].

Before fitting any models, we removed species from the analysis with any missing data, resulting in a dataset of 19 species. We adopted an information-theoretic approach to analyse the effects of social and environmental factors on non-reproductive and reproductive division of labour. PGLS models describing each possible iteration of specific hypotheses were fitted to the data. We used the corrected Akaike information criterion (AICc) to assess model fit and calculated small-sample AICc weight and ΔAICc (the difference in AICc between the model in question and the best fitting model) for each model. Models with ΔAICc > 7 were considered uninformative and were discarded [68]. As no model had an AICc weight more than 0.44 and the informative models for each hypothesis included between them all covariates, we do not report a single best model. We instead report relative importance and averaged parameter estimates from the set of informative models [68].

The averaged models were based on a single consensus phylogenetic tree (a strict consensus of 10 000 equally parimonious trees). Parameter estimates of the models are influenced by the phylogenetic estimate used and therefore are sensitive to other reconstructions [69]. To investigate the effects of phylogenetic uncertainty on our analysis, we fitted PGLS models on a dated sample of 1000 of the 10 000 most parsimonious trees from the PAUP* analysis of the MRP matrix. We selected only variables that had a cumulative AICc weight of more than 0.4 for these models. This allowed more accurate measurements of parameter estimates, which were generated as means from the sample of models, and of 95% phylogenetic uncertainty intervals [69].

## 3. Results

**Figure 1.**
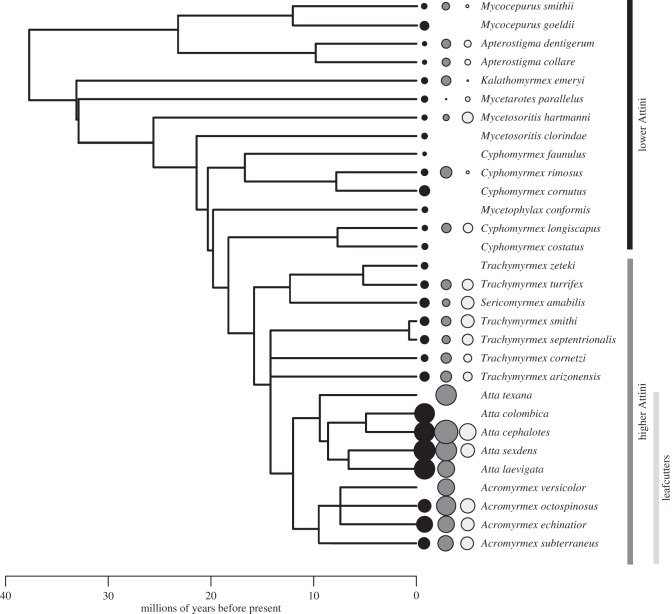
Distribution of colony size, worker size variation and queen–worker dimorphism on a phylogenetic supertree for the Attini (30 species). The full tree (electronic supplementary material, figure S2) was pruned to include only the species for which there were data on at least one trait and appeared in the phylogeny. Black circles are proportional to ln mean colony size, grey circles to the square root of worker size variation and white circles to ln queen–worker dimorphism. Branch lengths are proportional to time (Myr).

Mean worker size variation ranged from 0.23 to 64.37 (36 species), queen–worker dimorphism from 1.54 to 84.25% (30 species) and colony size from 16 to 6 × 10^6^ workers (43 species). The largest values for all these traits were found in the genera *Atta* and *Acromyrmex* (the leafcutter ants) (figure 1).

### (a) Phylogenetic reconstruction

The topology of our supertree (electronic supplementary material, figure S2) was broadly in agreement with the most recent molecular phylogeny for the Attini [70]. Clades that emerged as paraphyletic were *Cyphomyrmex* (with respect to *Mycetophylax conformis*) (node 7) and *Trachymyrmex* (with respect to *Sericomyrmex*) (node 12). None of these relationships are novel [70–72] and no novel clades were generated [73]. Furthermore, the supertree recovered the three clades of Attini defined by the nature of their fungal–agricultural system, i.e. the lower attines (which cultivate environmentally derived fungi), the higher attines excluding leafcutters (which engage in obligate fungal symbiosis but do not harvest fresh leaves) and the leafcutters (which engage in obligate fungal symbiosis and harvest fresh leaves) [31,70]. The mean rQS score over 10 000 bootstrap replicates of the tree was 0.282 and only three (nodes 52, 59 and 60) of the 60 nodes had a negative rQS score (reflecting more mismatches than matches in the source trees) (see the electronic supplementary material, table S3). We dated the root node (node 1) to 37.7 Ma, the node representing the origin of the higher Attini to 17.3 Ma and the origin of the leafcutters to 12 Ma. While this root estimate is 8.3–17.3 Myr younger than equivalent nodes on other molecular trees [70], the other values of the other nodes are within the confidence intervals (CIs) of previous estimates [70].

### (b) Determinants of non-reproductive division of labour

**Figure 2.**
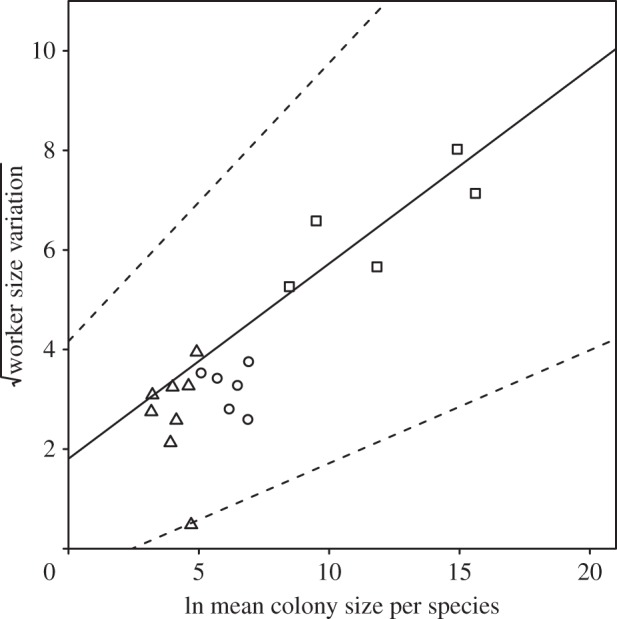
The relationship between ln mean colony size and square-root worker size variation in the 19 species of Attini for which colony size and worker size variation data were available; triangles represent the lower Attini, circles the higher Attini (excluding the leafcutter ants) and squares the leafcutter ants. Slope and intercept are taken from the phylogenetically controlled averaged model (table 1), and dotted lines are ±95% CIs from the same model.

Colony size was significantly positively correlated with worker size variation (table 1 and figure 2). All models featured colony size as a covariate and had a range of high *R*^2^ values (0.770–0.818), and colony size had a cumulative AICc weight of 1, showing its importance in all supported models. Furthermore, colony size was the only covariate in the averaged model to have CIs that did not include zero (table 1). The presence of queen–worker dimorphism, mean diurnal temperature range and precipitation seasonality in the averaged model suggest they have an effect on worker size variation; however, all three of these covariates had CIs that included zero (table 1). Models omitting colony size had no support (*w_i_* = 0 in both cases, electronic supplementary material, table S4*a*). These models were robust to phylogenetic uncertainty (table 1). Differences in mating systems among the Attini could have potentially confounded our analyses as species that were found to exhibit the largest colony sizes and worker size variation (leafcutter ants) are polyandrous [72]. To investigate this, we reanalysed our data including mating system as a dichotomous variable (0, monandrous and 1, polyandrous) in the models. We used all data on the presence and absence of polyandry from the literature and, for non-leafcutter ant species where data were not available, we assumed monandry (electronic supplementary material, table S1). We found the significance of the correlation between colony size and worker size variation to be unchanged while controlling for queen–worker dimorphism and mating system (for colony size, *β* = 0.271 (CI = 0.133, 0.509), *W* = 0.93, results from an averaged model).

**Table 1.** Averaged models describing effects of covariates on worker size variation, queen–worker dimorphism (where (*a*) and (*b*) represent models excluding and including worker size variation, respectively) and colony size in the Attini. Regression coefficients and CIs are reported from best (ΔAICc < 7) PGLS models from full candidate sets (see the electronic supplementary material, table S4*a*, *b* and *c*). Bold type indicates significant covariates. *β* = model averaged regression slope (95% CIs), *p*CI = 95% CI for the regression slope from 1000 models including parameters with *W* > 0.4 from a sample of 1000 equally likely trees; *W* = cumulative AICc weight over all models from the full candidate model set.

covariates	worker size variation	queen–worker dimorphism (a)	queen–worker dimorphism (b)	colony size
(intercept)	*β* = 1.806 (−0.554, 4.165), *p*CI ± 0.000	*β* = 1.659 (1.644, 1.675), *p*CI ± 0.031	*β* = 0.923 (−0.957, 2.802), *p*CI ± 0.010	*β* = 4.230 (23.954, 12.414), *p*CI ± 0.146
colony size	***β* = 0.392 (0.227, 0.559), *p*CI ± 0.000, *W* = 1.00**	***β* = 0.159 (0.042, 0.276), *p*CI ± 0.001, *W* = 0.85**	*β* = 0.135 (−0.030, 0.301), *p*CI ± 0.005, *W* = 0.60	—
worker size variation	—	—	*β* = 0.235 (−0.127, 0.598), *p*CI ± 0.011, *W* = 0.52	—
queen–worker dimorphism	*β* = 0.108 (−0.497, 0.713), *p*CI± 0.000, *W* = 0.48	—	—	—
mean diurnal temperature range	*β* = −0.015 (−0.035, 0.0058), *p*CI ± 0.000, *W* = 0.69	—	—	*β* = −0.021 (−0.071, 0.029), *p*CI ± 0.001, *W* = 0.40
isothermality	—	*β* = −0.004 (−0.044, 0.036), *W* = 0.28	*β* = −0.003 (−0.046, 0.038), *W* = 0.33	*β* = 0.022 (−0.126, 0.170), *p*CI ± 0.001, *W* = 0.50
temperature seasonality	—	—	—	—
precipitation seasonality	*β* = 0.013 (−0.017, 0.043), *p*CI ± 0.000, *W* = 0.041	*β* = 0.0009 (−0.014, 0.016), *W* = 0.28	*β* = 0.004 (−0.009, 0.016), *W* = 0.36	*β* = −0.015 (−0.210, 0.179), *p*CI ± 0.001, *W* = 0.62
isothermality × precipitation seasonality	—	—	—	*β* = 0.003 (−0.0002, 0.006), *W* = 0.13
latitude	—	—	—	*β* = 0.009 (−0.077, 0.096), *W* = 0.19

### (c) Determinants of reproductive division of labour

To complement the analysis of Fjerdingstad & Crozier [21], which found that colony size and worker size variation across 35 ant species were not significantly associated after controlling for queen–worker dimorphism, we first ran a model that included worker size variation as a covariate. This found no effect of colony size on queen–worker dimorphism. The resulting averaged model had only parameter estimates with CIs that included zero (table 1). The best fitting model set also captured less of the variation in queen–worker dimorphism than the models for worker size variation (*r*^2^ = 0.031–0.342). These analyses were robust to phylogenetic uncertainty (table 1). However, according to our VIF threshold (VIF for worker size variation = 4.80), colony size and worker size variation could not be in the model together. We therefore ran models omitting worker size variation, which showed colony size to be a positive predictor of queen–worker dimorphism (table 1). The effect was not as powerful as the effect of colony size on worker size variation, and the covariate was not universally shared in the most informative models (cumulative AICc weight = 0.85). Overall, therefore, we found a significant positive correlation between colony size and queen–worker dimorphism, but this result was weaker than the correlation of colony size with worker size variation. Moreover, it disappeared when worker size variation was included as a covariate, either because of shared variance or because worker size variation predicts queen–worker dimorphism better than colony size.

### (d) Environmental determinants of colony size

We found no significant correlations between colony size and any of the environmental variables tested (table 1). The *r*^2^ value of all models was low (range 0.001–0.211) and in all resulting average models the CIs of the covariates overlapped with zero.

## 4. Discussion

In agreement with the size–complexity hypothesis [4,6,12,13], our study shows that colony size is significantly positively correlated with measures of non-reproductive and reproductive division of labour in a tribe of ants. These findings provide novel support for the size–complexity hypothesis; we detected a strong relationship between colony size and worker size variation independent of the effects of queen–worker dimorphism, we controlled for environmental factors and we separated social complexity into component traits. Our results are also consistent with a recent study linking colony size with another predicted correlate of social complexity [4,6,12], namely divergence in queen and worker lifespans in the eusocial Hymenoptera [44]. In addition, our results strengthen the idea that group size and complexity are positively related in the evolution of other levels of complexity within the hierarchy of major transitions, such as the evolution of multicellularity [4,5,13,74].

We found no evidence for any effects of environmental factors on worker size variation, queen–worker dimorphism or colony size. Although colony size and primary productivity appear to be associated in ants, the relationship is nonlinear [23] and, in general, relationships between colony size, latitude and climatic variables vary considerably across ant taxa [75]. Therefore, the lack of effects of environmental factors in our study could have arisen because Attini are exceptions to the colony size–primary productivity relationship or because the study sampled species across the range of primary productivities where the relationship is approximately flat [23].

Our results suggest that colony size acts upon the two forms of division labour in different ways. Specifically, we found that the positive association between colony size and queen–worker dimorphism became non-significant when worker size variation was included, whereas the positive association between colony size and worker size variation remained significant in both the presence and absence of queen–worker dimorphism. If the two forms of division of labour responded to increasing colony size in the same way, we would have expected to see any combination of the two measures result in the absence of a positive association (due to very high colinearity). One plausible scenario that could account for our findings is non-simultaneous evolution of the two traits. A potential mechanism for this arises from an assumption of the size–complexity hypothesis, namely that the chance of any given worker attaining direct fitness falls as colonies evolve to become larger [4,6,12]. If so, this would lead workers’ inclusive fitness interests to coincide more closely with those of queens at larger colony sizes [4], because workers would be more strongly selected to maximize their fitness indirectly by aiding the direct reproduction of queens. Selection for worker size variation, which helps improve colony productivity [9,76], might then lead to even stronger selection for increased fecundity in queens and hence to greater queen–worker dimorphism. This hypothesis could be tested by investigating the order of trait divergence among worker size variation, queen–worker dimorphism and colony size, or by investigating the rates of evolutionary change of these traits.

An unexpected association from our results was a link between fungal–agricultural system and colony size. This was shown by the clustering of the three agricultural groups within the Attini, i.e. lower attines, higher attines (excluding leafcutter ants) and leafcutter ants, within the continuum of association between worker size variation and colony size (figure 2). To investigate this more formally, we examine the relationship between colony size and fungal–agricultural system. We find that colony size has a highly significant effect on agricultural system when treated as either a continuous variable (PGLS, *β* = 0.12, *p* < 0.001) or a categorical variable (univariate multinomial logistic regression, see the electronic supplementary material, Multinomial model analysis). Although it is not possible from current data to determine the evolutionary sequence of events, a possible scenario is that shifts in the fungal–agricultural system in the Attini act as ecological drivers permitting increases in colony size and that these then lead to increases in the complexity of division of labour proposed by the size–complexity hypothesis and detected by our analysis.

As phylogenetic reconstructions and large datasets of social and environmental trait data become increasingly available, studies like the present one that combine the power of phylogenetically controlled analyses with the rich social and ecological diversity of eusocial insects will help test the size–complexity hypothesis in additional taxa and, more generally, investigate further how social and environmental factors influence the evolution of social complexity and division of labour within societies.

## Supplementary Material

Ferguson-Gowetal_esm
