# Supplementary material for: Colony size predicts division of labour in attine ants
Source: Proc Biol Sci. 2014 Oct 22;281(1793):20141411. doi: 10.1098/rspb.2014.1411 (PMC4173680; doi:10.1098/rspb.2014.1411)
Supplement: Ferguson-Gowetal_esm [file rspb20141411supp1.pdf]

Colony size predicts division of labour in Attine ants: Electronic supplementary material

Henry Ferguson-Gow[1,2] *†, Seirian Sumner[2,3], Andrew F. G. Bourke[1] and Kate E. Jones[2,4]*

[1] School of Biological Sciences, University of East Anglia, Norwich Research Park, Norwich, Norfolk, NR4 7TJ, United Kingdom

[2] Institute of Zoology, Zoological Society of London, Regent's Park, London, NW1 4RY, United Kingdom

[3] Current address: School of Biological Sciences, University of Bristol, Woodland Road, Bristol, BS8 1UG, United Kingdom

[4] Centre for Biodiversity and Environment Research, Department of Genetics, Evolution and Environment, University College London, Gower Street, London, WC1E 6BT, United Kingdom

† Current address: Centre for Biodiversity and Environment Research, Department of Genetics, Evolution and Environment, University College London, Gower Street, London, WC1E 6BT, United Kingdom

* Corresponding author: h.ferguson-gow@ucl.ac.uk and kate.e.jones@ucl.ac.uk

Table S1. Life history and environmental traits and data sources for 57 species of Attini. W and Q represent Worker and Queen, respectively. Values for colony size, worker and queen head-widths represent per-species means (in mm) calculated by averaging the mean value from each observation weighted by its sample size; Σns is the sum of all the sample sizes of the observations contributing to the per-species mean for each trait (colony size, worker and queen head width) (assumed to be 1 where observation sample sizes were not given); worker size variation is the coefficient of variation in worker head-widths; queen-worker dimorphism is percentage change between mean queen and worker head-width; and mean diurnal temperature range, isothermality, temperature seasonality and precipitation seasonality are taken from BioClim (www.worldclim.org/bioclim). Taxonomy follows Bolton World Catalogue (www.antweb.org), and - denotes missing data.

Species	Mean Colony Size	Σns Colony Size	Mean W Head-Width	Σns W Head-Width	Mean Q Head-Width	Σns Q Head-Width	W Size Variation	Q-W Dimorphism	Polyandrous	Mean Diurnal Temp. Range	Isothermality	Temp Seasonality	Precipitation Seasonality	N Geolocated Points	Data Source
Acromyrmex ameliae	-	-	1.1	30	1.4	21	27.27	24	-	130	69	1681	85	6	[1]
Acromyrmex echinatior	137500	5	1.87571	826	2.58	11	32.05	31.61	Yes	100.02	74.32	745.85	80.9	42	[2-7]
Acromyrmex insinuator	100	1	-	-	2.325	27	-	-	Yes	72	72	724	60	13	[3, 4, 7, 9, 10]
Acromyrmex octospinosus	13375	5	1.80769	581	2.9	5	43.33	46.41	Yes	84	75.6	717.48	39.54	71	[2-4, 6, 11-14]
Acromyrmex subterraneus	4766.2	5	1.58767	16	2.15	17	27.71	30.09	-	130	69	1681	85	4	[1, 15, 16]
Acromyrmex versicolor	-	-	2.0692	5	-	-	33.36	-	Yes	162.56	46.22	6971.11	53.67	18	[1, 2, 15, 17]
Apterostigma collare	23.98	128	0.8695	3	0.91	2	7.56	4.55	No	90.13	77.19	688.05	32.93	165	[3, 15, 19-21]
Apterostigma dentigerum	25	1	1.069	3	1.14	1	9.53	6.43	-	89.3	76.99	715.96	30.42	93	[3, 15]
Apterostigma mayri	20	25	-	-	-	-	-	-	-	70	72.5	706	58.5	10	[12]
Apterostigma sp. 2	25	1	-	-	1.09	1	-	-	-	72	72	724	60	2	[3]
Apterostigma urichii	23	1	-	-	-	-	-	-	-	95	83.33	470.67	46.33	3	[22]
Atta cephalotes	3000000	1	1.98714	949	4.88	5	64.37	84.25	Yes	90.11	77.19	649.9	30.43	214	[3, 6, 15, 23, 24]
Atta colombica	2266666.67	5	-	-	4.31	5	-	-	-	76.58	74.21	680.68	56.68	30	[3, 12, 25, 26]
Atta laevigata	3500000	1	2.87929	7	-	-	33.5	-	Yes	108.2	74.6	1095.2	62	5	[15, 25, 27, 28]
Atta sexdens	6000000	1	2.46363	1016	3.62	4	50.93	38.16	Yes	84.67	78	667.33	79	6	[2, 3, 15, 25]
Atta texana	-	-	2.304	3	-	-	48.98	-	Yes	132	39	7287	16	7	[15, 29]
Atta vollenweideri	5500000	1	2.99467	3	4.91	2	53.96	48.61	-	122.33	54.67	3465	46.33	5	[15, 25, 26]
Cyphomyrmex cornutus	2021.75	4	-	-	-	-	-	-	-	90.18	77.01	671.58	29.58	73	[15, 30]
Cyphomyrmex costatus	96.5	53	-	-	0.58	5	-	-	-	85.96	76.29	645.87	45.09	48	[3, 12, 15, 31]
Cyphomyrmex faunulus	16	1	-	-	-	-	-	-	-	92	86	338	20	2	[15, 22]
Cyphomyrmex longiscapus	54.13	203	0.65778	93	0.746667	25	10.53	12.66	No	85.24	75.36	637.48	49.14	42	[15, 21, 32, 33]
Cyphomyrmex muelleri	-	-	0.63833	78	0.7	23	6.68	9.22	-	-	-	-	-	-	[33]
Cyphomyrmex rimosus	136.75	69	0.632	4	0.646333	3	15.58	2.24	-	93.68	74.44	1005.29	45.61	259	[3, 12, 15, 34]
Kalathomyrmex emeryi	100	1	0.82467	10	0.8375	2	10.69	1.54	-	97.75	65.75	1826	61.75	5	[3, 15]
Mycetagroicus cerradensis	373	2	0.8705	3	-	-	3.98	-	-	126	69	671.73	44.92	5	[15, 3]
Mycetagroicus inflatus	-	-	0.715	2	-	-	0.99	-	-	-	-	-	-	-	[36]
Mycetarotes carinatus	-	-	-	-	0.88	1	-	-	-	-	-	-	-	-	[58]
Mycetarotes parallelus	110.57	13	0.9175	4	0.95	3	0.23	3.48	-	126.91	56.91	3284.68	20.41	21	[3, 15, 37]
Mycetophylax conformis	72.25	3	-	-	-	-	-	-	-	-	-	-	-	-	[15, 38]
Mycetophylax morschi	72.25	4	-	-	-	-	-	-	-	-	-	-	-	-	[15, 38]
Mycetophylax simplex	191.94	7	-	-	-	-	-	-	-	-	-	-	-	-	[15, 38, 39]
Mycetosoritis clorindae	70.5	2	-	-	-	-	-	-	-	92	48	3306	9	3	[40]
Mycetosoritis explicate	-	-	0.8	2	0.93	1	14.52	15.03	-	-	-	-	-	-	[41, 15]
Mycetosoritis hartmanni	50	2	0.6375	4	0.77	3	4.53	18.83	-	110.4	45.5	5210.9	36.8	10	[3, 15]
Mycocepurus castrator	1	2	-	-	0.6	16	-	-	-	116	63	2405	71	6	[42]
Mycocepurus goeldii	678.86	7	-	-	0.78	2	-	-	-	91.63	76.88	945.63	50.44	16	[15, 42-44, 47]
Mycocepurus obsoletus	-	-	-	-			-	-	-	87	76	594	71	2	[15, 43]
Mycocepurus smithii	62.63	162	0.6275	4	0.614	1	6.65	2.17	-	89.83	78.07	779.41	27	46	[15, 39, 44-47]
Myrmicocrypta bucki	-	-	0.64333	4	-	-	3.91	-	-	105	73	1014	45	3	[48]

Myrmicocrypta camargoi	-	-	0.75333	4	1.09	2	5.53	36.53	-	117	63	2333	60	5	[48]
Myrmicocrypta ednaella	86.78	35	-	-	-	-	-	-	No	88.3	78.06	642.76	46.81	67	[12, 21, 34]
Myrmicocrypta erectapilosa	-	-	0.59333	4	0.71	1	5.42	17.9	-	83	81	446	47	5	[48]
Sericomyrmex amabilis	972.67	12	0.9335	10	1.255	6	6.74	29.38	No	88.26	76.74	708.61	32.36	334	[2, 3, 12, 15, 20, 49]
Trachymyrmex arizonensis	1000	1	1.15333	6	1.285	2	14.11	10.8	-	172.5	49.44	6419.72	68.72	17	[15, 50, 52]
Trachymyrmex carinatus	100	1	0.94767	5	1.246	2	13.63	27.2	-	166	51	5626	113	3	[15, 52]
Trachymyrmex cornetzi	161.83	62	0.975	5	1.06	2	12.46	8.35	No	87.96	76.58	721.07	33.04	147	[2, 3, 15, 49, 57]
Trachymyrmex desertorum	-	-	1.0584	5	1.375	2	16.71	26.02	-	-	-	-	-	-	[15, 52]
Trachymyrmex isthmicus	100.67	43	-	-	-	-	-	-	-	81.5	75.29	722.14	39.86	28	[52]
Trachymyrmex jamaicensis	525	2	1.37033	3	1.65	2	5.23	18.52	-	91.5	54.75	2696.25	48.25	7	[15, 52]
Trachymyrmex nogalensis	-	-	1.34225	4	1.425	2	16.57	5.98	-	-	-	-	-	-	[52]
Trachymyrmex pomonae	183	1	0.865	2	1.066667	3	13.9	20.88	-	178	50	6516	70	3	[52]
Trachymyrmex septentrionalis	474.7	93	0.967	552	1.160667	2	7.87	18.2	-	122.93	48	4974.6	44.27	17	[15, 52-55]
Trachymyrmex smithi	652.5	4	1.37329	6	1	2	10.76	31.46	-	167.5	55.5	4822	88.5	5	[15, 52]
Trachymyrmex sp. 3	1000	1			1.22	5	-	-	-	72	72	724	60	3	[3]
Trachymyrmex turrifex	300	1	0.9535	4	1.15	2	11.72	18.68	-	173	51	5626	113	4	[15, 52]
Trachymyrmex urichii	-	-	-	-	-	-	-	-	-	109.5	78	586.5	57	3	[15, 56]
Trachymyrmex zeteki	146.78	136	-	-	1.29	5	-	-	No	80.55	74.62	708.28	46.76	30	[2, 3, 15, 20, 49, 51]

Table S1 Data Sources.

1. De Souza DJ, Soares IMF, Della Lucia TMC. 2007 *Acromyrmex ameliae* sp. n. (Hymenoptera: Formicidae): A new social parasite of leaf-cutting ants in Brazil. *Insect Sci.* **14**, 251-257
2. Dijkstra MB, Boomsma JJ. 2008 Sex allocation in fungus-growing ants: worker or queen control without symbiont-induced female bias. *Oikos* **117**, 1892-1906
3. Baer B, Dijkstra MB, Mueller UG, Nash DR, Boomsma JJ. 2008 Sperm length evolution in the fungus-growing ants. *Behav. Ecol.* **20**, 38-45
4. Schultz TR, Bekkevold D, Boomsma JJ. 1998 *Acromyrmex insinuator* new species: an incipient social parasite of fungus-growing ants. *Insectes Soc.* **45**, 457-471
5. Hughes WO, Sumner S, Van Borm S, Boomsma JJ. 2003 Worker caste polymorphism has a genetic basis in *Acromyrmex* leaf-cutting ants. *Proc. Natl. Acad. Sci. USA* **100**, 9394-9397
6. Dijkstra MB, Nash DR, Boomsma JJ. 2005 Self-restraint and sterility in workers of *Acromyrmex* and *Atta* leafcutter ants. *Insectes Soc.* **52**, 67-76
7. Sumner S, Aanen DK, Delabie J, Boomsma JJ. 2004 The evolution of social parasitism in *Acromyrmex* leaf-cutting ants: a test of Emery's rule. *Insectes Soc.* **51**, 37-42
8. Bekkevold D, Freydenberg J, Boomsma JJ. 1999 Multiple mating and facultative polygyny in the Panamanian leafcutter ant *Acromyrmex echinatior*. *Behav. Ecol. Sociobiol.* **46**, 103-109
9. Sumner S, Hughes WOH, Pedersen JS, Boomsma JJ. 2004 Ant parasite queens revert to mating singly. *Nature* **428**, 35-36
10. Bekkevold D, Boomsma JJ. 2000 Evolutionary transition to a semelparous life history in the socially parasitic ant *Acromyrmex insinuator*. *J. Evol. Biol.* **13**, 615-623
11. Wetterer JK, Gruner DS, Lopez JE. 1998 Foraging and nesting ecology of *Acromyrmex octospinosus* (Hymenoptera : Formicidae) in a costa Rican tropical dry forest. *Fla. Entomol.* **81**, 61-67
12. Murakami T, Higashi S, Windsor D. 2000 Mating frequency, colony size, polyethism and sex ratio in fungus-growing ants (Attini). *Behav. Ecol. Sociobiol.* **48**, 276-284
13. Muscedere ML, Berglund JL, Traniello JFA. 2010 Polymorphism and division of labor during foraging cycles in the leaf-cutting ant *Acromyrmex octospinosus* (Formicidae; Attini). *J. Insect Behav.* **24**, 94-105
14. Boomsma JJ, Fjerdingstad EJ, Frydenberg J. 1999 Multiple paternity, relatedness and genetic diversity in *Acromyrmex* leaf-cutter ants. *Proc. R. Soc. B* **266**, 249-254
15. Antweb. 2012 http://www.antweb.org Accessed 17/06/2012.
16. Camargo RS, Forti LC, de Andrade APP, de Matos CAO, Lopes JFS. 2005 Morphometry of the sexual forms of *Acromyrmex subterraneus brunneus* Forel, 1911 (Hym., Formicidae) in queenright and queenless laboratory colonies. *J. Appl. Entomol.* **129**, 347-351
17. Reichardt AK, Wheeler DE. 1998 Multiple mating in the ant *Acromyrmex versicolor*: a case of female control. *Behav. Ecol. Sociobiol.* **38**, 219-225
18. Rissing SW, Johnson RA, Pollock GB. 1986 Natal nest distribution and pleometrosis in the desert leaf-cutter ant, *Acromyrmex versicolor* (Pergande)(Hymenoptera: Formicidae). *Psyche* **93**, 177-186
19. Pitts-Singer TL, Espelie KE. 2007 Nest demographics and foraging behavior of *Apterostigma collare* Emery (Hymenoptera, Formicidae) provide evidence of colony independence. *Insectes Soc.* **54**, 310-318
20. Villesen P, Boomsma JJ. 2003 Patterns of male parentage in the fungus-growing ants. *Behav. Ecol. Sociobiol.* **53**, 246-253
21. Villesen P, Gertsch PJ, Frydenberg J, Mueller U, Boomsma JJ. 1999 The Evolutionary transition from single to multiple mating in fungus-growing ants. *Mol. Ecol.* **8**, 1819-1825.
22. Sanhudo CED, Izzo TJ, Brandão CRF. 2008 Parabiosis between basal fungus-growing ants (Formicidae, Attini). *Insectes Soc.* **55**, 296-300
23. Abramowski D, Currie CR, Poulsen M. 2011 Caste specialization in behavioral defenses against fungus garden parasites in *Acromyrmex octospinosus* leaf-cutting ants. *Insectes Soc.* **58**, 65-75
24. Wetterer JK. 1991 Allometry and the geometry of leaf-cutting in *Atta cephalotes*. *Behav. Ecol. and Sociobiol.* **29**, 347-335
25. Hölldobler B, Wilson EO. 1990 *The Ants*, Belknap Press.
26. Jonkman J. 1980 Average vegetative requirement, colony size and estimated impact of *Atta vollenweideri* on cattle-raising in Paraguay. *Zeitschrift fur Angewandte Entomologie* **89**, 135-143
27. Moreira DD, Bailez AM, Erthal Jr M, Bailez O, Carrera MP, Samuels RI. 2010 Resource allocation among worker castes of the leaf-cutting ants *Acromyrmex subterraneus subterraneus* through trophallaxis. *J. Insect Physiol,* **56**, 1665-1670
28. Hernández JV, Cabrera A, Jaffe K. 1999 Mandibular gland secretion in different castes of the leaf-cutter ant *Atta laevigata*. *J. Chem. Ecol.* **25**, 2433-2444
29. Moser JC. 2006 Complete excavation and mapping of a Texas leafcutting ant nest. *Ann. Entomol. Soc. Am.* **99**, 891-897
30. Adams RMM, Longino JT. 2007 Nesting biology of the arboreal fungus-growing ant *Cyphomyrmex cornutus* and behavioral interactions with the social-parasitic ant *Megalomyrmex mondabora*. *Insectes Soc.* **54**, 136-143
31. Kweskin MP. 2004 Jigging in the fungus-growing ant *Cyphomyrmex costatus* : a response to collembolan garden invaders? *Insectes Soc.* **51**, 158-162
32. Mueller UG, Wcislo WT. 1998 Nesting biology of the fungus-growing ant *Cyphomyrmex longiscapus* Weber (Attini, Formicidae) *Insectes Soc.* **45**, 181-189
33. Schultz TR, Solomon SA, Mueller UG, Villesen P, Boomsma JJ, Adams RMM, Norden B. 2002 Cryptic speciation in the fungus-growing ants *Cyphomyrmex longiscapus* Weber and *Cyphomyrmex muelleri* Schultz and Solomon, new species (Formicidae, Attini). *Insectes Soc.* **49**, 331-343
34. Murakami T, Higashi S. 1997 Social organization in two primitive attine ants, *Cyphomyrmex rimosus* and *Myrmicocrypta ednaella*, with reference to their fungus substrates and food sources. *J. Ethol.* **15**, 17-25
35. Solomon SE, Lopes CT, Mueller UG, Rodrigues A, Sosa-Calvo J, Schultz TR, Vasconcelos HL. 2011 Nesting biology and fungiculture of the fungus-growing ant, *Mycetagroicus cerradensis*: new light on the origin of higher attine agriculture. *J. Insect Sci.* **11**, 12
36. Brandão CRF, Mayhé-Nunes AJ. 2008 A new species of the fungus-farming ant genus *Mycetagroicus* Brandão & Mayhé-Nunes (Hymenoptera, Formicidae, Attini). *Rev. Bras. Entomol.* **52**, 349-352
37. Solomon SE, Mueller UG, Schultz TR, Currie CR, Price SL, Oliveira da Silva-Pinhati AC, Bacci M, Vasconcelos HL. 2004 Nesting biology of the fungus growing ants *Mycetarotes* Emery (Attini, Formicidae). *Insectes Soc.* **51**, 333-338
38. Cardoso DC, Cristiano MP, Tavares MG. 2011 Methodological remarks on rearing basal Attini ants in the laboratory for biological and evolutionary studies: overview of the genus Mycetophylax. *Insectes Soc.* **58**, 427-430
39. Diehl-Fleig E, Diehl E. 2007 Nest architecture and colony size of the fungus-growing ant *Mycetophylax simplex* Emery, 1888 (Formicidae, Attini). *Insectes Soc.* **54**, 242-247
40. Mueller UG, Ortiz A, Bacci M. 2010 Planting of fungus onto hibernating workers of the fungus-growing ant *Mycetosoritis clorindae* (Attini, Formicidae). *Insectes Soc.* **57**, 209-215
41. Sosa-Calvo J, Brady SG, Schultz TR. 2009 The gyne of the enigmatic fungus-farming ant species *Mycetosoritis explicata*. *J. Hymenoptera Res.* **18**, 113-120

42. Rabeling C, Bacci M. 2010 A new workerless inquiline in the Lower Attini (Hymenoptera: Formicidae), with a discussion of social parasitism in fungus-growing ants. *Syst. Entomol.* **35**, 379-392
43. Mackay WP, Maes JM, Fernandez PR, Luna G. 2004 The ants of North and Central America: the genus *Mycocepurus* (Hymenoptera : Formicidae). *J. Insect Sci.* **4**, 27
44. Rabeling C, Verhaagh,M, Engels W. 2007 Comparative study of nest architecture and colony structure of the fungus-growing ants, *Mycocepurus goeldii* and *M. smithii*. *J. Insect Sci.* **7**, 40
45. Fernández-Marín H, Zimmerman JK, Wcislo WT, Rehner SA. 2005 Colony foundation, nest architecture and demography of a basal fungus-growing ant, *Mycocepurus smithii* (Hymenoptera, Formicidae). *J. Nat. Hist.* **39**, 1735-1743. (doi:10.1080/00222930400027462).
46. Himler AG, Caldera EJ, Baer BC, Fernandez-Marin H, Mueller UG. 2009 No sex in fungus-farming ants or their crops. *Proc. R. Soc. B* **276**, 2611-2616
47. Rabeling C, Lino-Neto J, Cappellari SC, Dos-Santos IA, Mueller UG, Bacci Jr M. 2009 Thelytokous parthenogenesis in the fungus-gardening ant *Mycocepurus smithii* (Hymenoptera: Formicidae). *Plos One* **4**, 1-10
48. Sosa-Calvo J, Schultz TR. 2010 Three Remarkable New fungus-growing ant species of the genus *Myrmicocrypta* (Hymenoptera: Formicidae), with a reassessment of the characters that define the genus and its position within the Attini. *Ann. Entomol. Soc. Am.* **103**, 181-195
49. Villesen P, Murakami T, Schultz TR. 2002 Identifying the transition between single and multiple mating of queens in fungus-growing ants. *Proc. R. Soc. B* **269**, 1541-1548
50. LaPolla JS, Mueller UG, Seid M, Cover SP. 2002 Predation by the army ant *Neivamyrmex rugulosus* on the fungus-growing ant *Trachymyrmex arizonensis*. *Insectes Soc.* **49**, 251-256
51. Pérez-Ortega B, Fernández-Marín H, Loiácono MS, Galgani P, Wcislo WT. 2010 Biological notes on a fungus-growing ant, *Trachymyrmex* cf. *zeteki* (Hymenoptera, Formicidae, Attini) attacked by a diverse community of parasitoid wasps (Hymenoptera, Diapriidae). *Insectes Soc.* **57**, 317-322
52. Rabeling C, Cover SP, Johnson RA, Mueller UG. 2007 A review of the North American species of the fungus-gardening ant genus *Trachymyrmex* (Hymenoptera: Formicidae). *Zootaxa* **1664**, 1-53
53. Bershers SN, Traniello JF. 1994 The adaptiveness of worker demography in the attine ant *Trachymyrmex septentrionalis*. *Ecology* **75**, 763-775
54. Seal JN, Tschinkel WR. 2008 Food limitation in the fungus-gardening ant,*Trachymyrmex septentrionalis*. *Ecol. Entomol.* **33**, 597-607
55. Seal JN, Tschinkel WR. 2006 Colony productivity of the fungus-gardening ant *Trachymyrmex septentrionalis* (Hymenoptera : Formicidae) in a Florida pine forest. *Ann. Entomol. Soc. America* **99**, 673-682
56. Jaffe K, Villegas G. 1985 On the communication systems of the fungus-growing ant *Trachymyrmex urichi*. *Insectes Soc.* **32**, 257-274
57. Kaspari M. (1996) Worker size and seed selection by harvester ants in a neotropical forest. *Oecologia* **105**, 397-404
58. Mayhé-Nunes, AJ & Lanziotti, AM (2004) Description of the female of *Mycetarotes carinatus* (Hymenoptera:Formicidae). *Rev. Biol. Trop.* **52**, 109-114

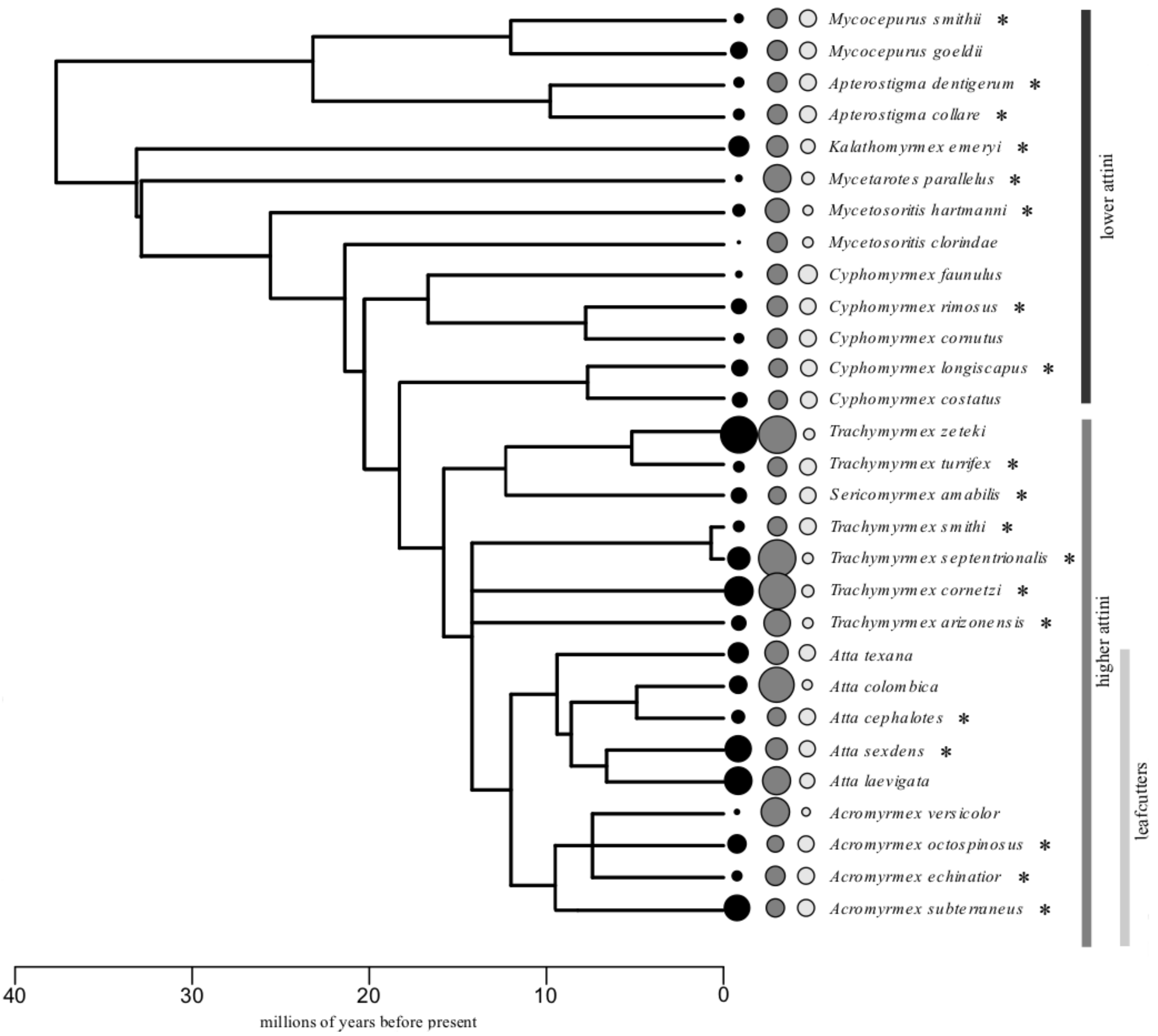

Figure S1. Distribution of precipitation seasonality, mean temperature fluctuation and isothermality on a phylogenetic supertree of the Attini (29 species). The full tree (electronic supplementary material, figure S2) was pruned to include only the species for which there were data on at least one trait and appeared in the phylogeny. Black, grey and white circles are proportional to precipitation seasonality, mean temperature fluctuation and isothermality, respectively. * denotes species used in the final analysis. Branch lengths are proportional to time (millions of years).

Table S2. Phylogenetic sources for the Attini supertree.

**Reference**	**Figure number**	**Data type**	**No. Attini species**
Bacci Jr et al 2009	1	mtDNA + tRNA	13
	2	nDNA	13
	3	mtDNA + nDNA	13
Brandao and Mayhé-Nunes 2007	2	Morphology	32
Moreau et al 2006	1	nDNA + mtDNA	4
Schultz and Brady 2008	1	nDNA + mtDNA	65
Shultz and Meier 1995	3	Larval morphology	51
Sumner et al 2004	1	mtDNA	19
Villesen et al 2004	6	mtDNA	12
Wetterer et al 1998	1	mtDNA + tRNA	14
	2	Amino acid sequence + morphology	14

Table S2 references.

Bacci Jr M, Solomon SE, Mueller UG, Martins VG, Carvalho AO, Vieira LG, Silva-Pinhati, ACO. 2009 Phylogeny of leafcutter ants in the genus *Atta* Fabricius (Formicidae: Attini) based on mitochondrial and nuclear DNA sequences. *Mol. Phylogenet. Evol.* **51**, 427-437.

Brandao CRF, Mayhé-Nunes AJ. 2007 A phylogenetic hypothesis for the Trachymyrmex species groups, and the transition from fungus-growing to leaf-cutting in the Attini. *Mem. Am. Entomol. Inst.* **80**, 72-88.

Moreau CS, Bell CD, Vila R, Archibald SB, Pierce NE. 2006 Phylogeny of the ants: diversification in the age of angiosperms. *Science* **312**, 101-104.

Schultz TR, Brady SG. 2008 Major evolutionary transitions in ant agriculture. *Proc. Natl. Acad. Sci. USA* **105**, 5435-5440.

Schultz TR, Meier R. 1995 A phylogenetic analysis of the fungus-growing ants (Hymenoptera: Formicidae: Attini) based on morphological characters of the larvae. *Syst. Entomol.* **20**, 337-370.

Sumner S, Aanen DK, Delabie J, Boomsma JJ. 2004 The evolution of social parasitism in Acromyrmex leaf-cutting ants: a test of Emery's rule. *Insectes Soc.* **51**, 37-42.

Villesen P, Mueller UG, Schultz TR, Adams RM, Bouck AC. 2004 Evolution of ant-cultivar specialization and cultivar switching in Apterostigma fungus-growing ants. *Evolution* **58**, 2252-2265.

Wetterer JK, Schultz TR, Meier R. 1998 Phylogeny of fungus-growing ants (Tribe Attini) based on mtDNA sequence and morphology. *Mol. Phylogenet. Evol.* **9**, 42-47.

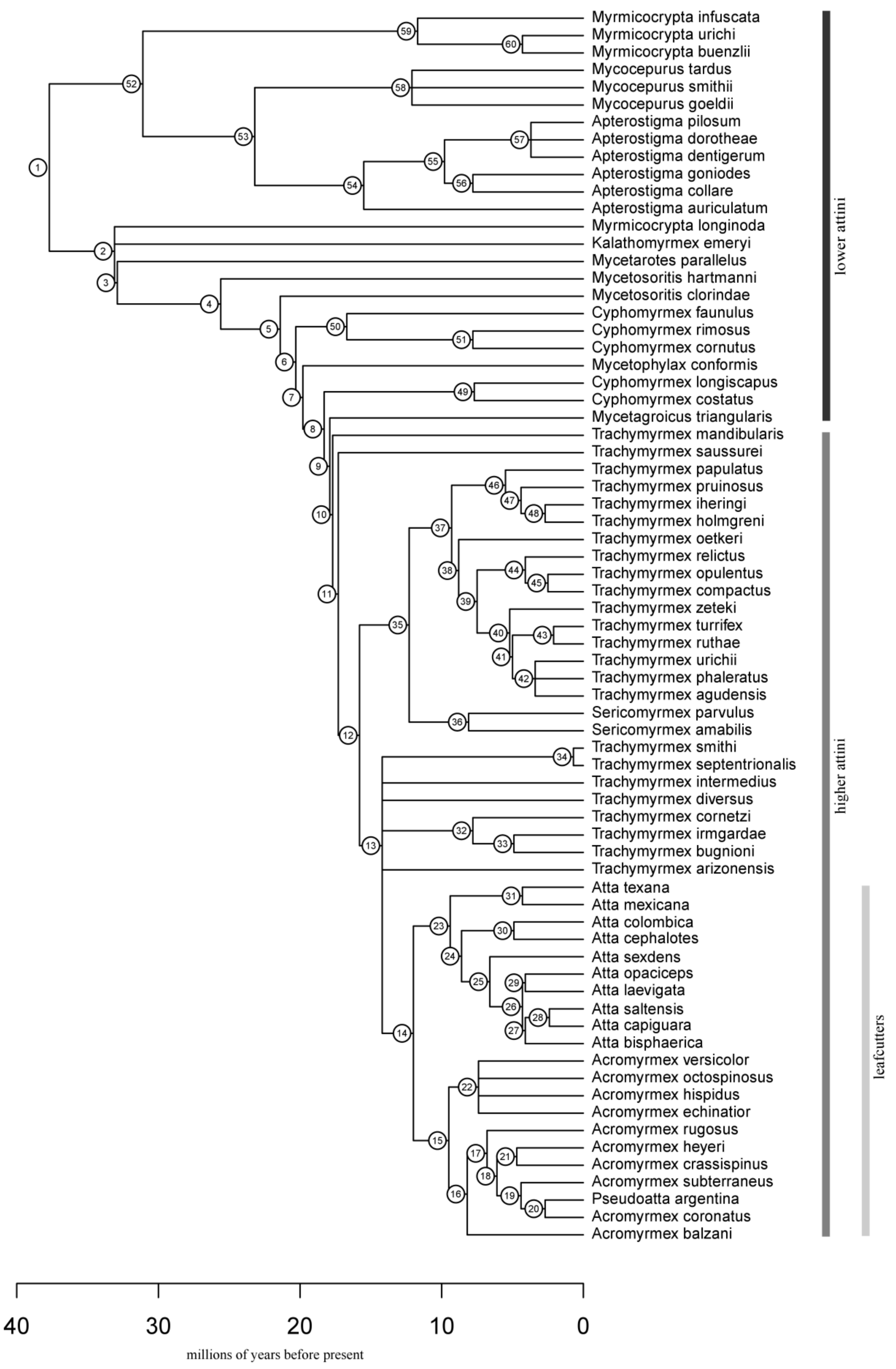

Figure S2. A phylogenetic supertree for 71 species of the Attini. Numbers on nodes are arbitrary numbers. Branch lengths are proportional to time (millions of years). See table S3 for nodal support values.

Table S3. Reduced qualitative support (rQS) scores for the Attini supertree (figure S1). rQS values range between 1 and -1 where 1 indicates full agreement for a node in the source trees, and -1 full disagreement.

Node #	rQS value	Node #	rQS value	Node #	rQS value
1	0.83	21	0.08	41	0.00
2	0.75	22	0.42	42	0.00
3	0.75	23	0.50	43	0.00
4	0.67	24	0.50	44	0.00
5	0.67	25	0.00	45	0.00
6	0.67	26	0.00	46	0.00
7	0.67	27	0.08	47	0.00
8	0.58	28	0.08	48	0.00
9	0.58	29	0.00	49	0.00
10	0.58	30	0.33	50	0.33
11	0.58	31	0.08	51	0.33
12	0.58	32	0.17	52	-0.83
13	0.33	33	0.00	53	0.25
14	0.50	34	0.08	54	0.42
15	0.33	35	0.17	55	0.25
16	0.83	36	0.33	56	0.25
17	0.42	37	0.00	57	0.01
18	0.17	38	0.00	58	0.25
19	0.17	39	0.00	59	-0.17
20	0.17	40	0.00	60	-0.17

Table S4. All models with ΔAICc < 7 describing the predictors of a) worker size variation, b) queen-worker dimorphism and c) colony size in the Attini. Regression slope estimates from phylogenetic least squares (PGLS) models are reported.

Table S4a.

**Intercept**	**Precipitation seasonality**	**Mean diurnal temperature fluctuation**	**log Colony size**	**Queen-worker dimorphism**	**Degrees of freedom**	**Log likelihood**	**AICc**	**ΔAICc**	**AICc weight**
2.342		-0.011	0.394		3	-23.97	55.7	0.68	0.19
1.027			0.401		2	-25.83	56.4	1.43	0.13
2.563	0.020	-0.020	0.347		4	-22.73	56.5	1.57	0.12
2.253	0.018	-0.020	0.353	0.152	5	-20.70	56.9	1.88	0.12
0.627	0.003		0.430	0.028	4	-22.79	56.9	1.93	0.10
0.832	0.005		0.391		3	-24.85	57.4	2.43	0.08

Table S4b.

**Intercept**	**Precipitation seasonality**	**Isothermality**	**log Colony size**	**Degrees of freedom**	**Log likelihood**	**AICc**	**ΔAICc**	**AICc weight**
0.912			0.154	2	-20.45	45.7	0	0.44
1.358		-0.007	0.168	3	-19.79	47.3	1.64	0.20
0.911	-0.001		0.159	3	-19.87	47.5	1.8	0.18
1.637				1	-23.84	49.9	4.26	0.05
1.291	0.008			2	-22.75	50.3	4.64	0.04
1.536	-0.002	-0.009	0.174	4	-19.76	50.6	4.93	0.04
0.681		0.014		2	-22.94	50.7	5.02	0.04
0.253	0.007	0.016		3	-22.34	52.4	6.74	0.02

Table S4c.

**Intercept**	**Precipitation seasonality**	**Mean diurnal temperature fluctuation**	**Isothermality**	**Lattitude**	**Precipitation seasonality * isothermality**	**Degrees of freedom**	**Log Likelihood**	**AICc**	**ΔAICc**	**AICc weight**
1.040			0.056			2	-61.93	128.4	0	0.17
6.658	0.044	-0.035				3	-60.73	128.6	0.18	0.16
4.245	0.015					2	-62.04	128.6	0.23	0.15
7.291	-0.173		-0.055		0.003	4	-59.81	129.5	1.14	0.10
4.976		-0.002				2	-62.57	129.7	1.29	0.09
1.437	0.011		0.044			3	-61.67	130.4	2.05	0.06
1.249		-0.002	0.056			3	-61.91	130.9	2.54	0.05
0.926			0.057	0.006		3	-61.92	130.9	2.55	0.05
4.240	0.015			0.002		3	-62.04	131.2	2.8	0.04
5.520	0.040	-0.032	0.015			4	-60.69	131.3	2.91	0.04
6.663	0.045	-0.035		0.007		4	-60.71	131.3	2.96	0.04
7.581	-0.202		-0.062	0.037	0.003	5	-59.36	131.7	3.34	0.03
4.992		-0.002		-0.005		3	-62.56	132.2	3.84	0.03

Multinomial Model Analysis

We categorised species by their agricultural system as follows: lower Attines (lower agriculture), higher Attines, excluding leafcutters (higher agriculture), and leafcutter ants (leafcutting agriculture). These classifications can be seen on figure 1, supplementary material figures S1 and S2. Using the fungal-agricultural system as the dependent variable and ln mean colony size as the independent variable, we fitted a univariate, multinomial logistic regression model using the R package MCMCglmm [1]. We used non-informative priors with a low degree of belief across all parameters, and ran the model for 6,000,000 generations, sampling every 1000th generation and discarding the first 25% of samples as burnin. We visually inspected the trace output to ensure model convergence and proper mixing, and made sure effective sample sizes were large enough to ensure robust parameter estimates.

We found strong evidence that the degree of fungal-agricultural system is predicted by colony size. Specifically, larger colony sizes are associated with a higher probability of higher agriculture versus lower agriculture (expected log-odds change per unit increase in ln colony size=1.21, CI_{95}=2.34, pMCMC=0.02), and leafcutter agriculture versus lower agriculture (expected log-odds change per unit increase in ln colony size=4.71, CI_{95}=7.19, pMCMC<<0.001).

1. Hadfield J.D. 2010 MCMC methods for multi-response generalized linear mixed models: the MCMCglmm R package. *Journal of Statistical Software* **33**(2), 1-22.
